# Influence of the Modifier Type and its Concentration on Electroosmotic Flow of the Mobile Phase in Pressurized Planar Electrochromatography

**DOI:** 10.1007/s10337-014-2689-6

**Published:** 2014-06-01

**Authors:** Aneta Hałka-Grysińska, Paweł W. Płocharz, Andrzej Torbicz, Ewa Skwarek, Władysław Janusz, Tadeusz H. Dzido

**Affiliations:** 1 Department of Physical Chemistry, Medical University of Lublin, Lublin, Poland; 2 Department of Cosmetology, Faculty of Physical Education and Sport in Biala Podlaska, Jozef Pilsudski University of Physical Education in Warsaw, Biala Podlaska, Poland; 3 Department of Radiochemistry and Colloid Chemistry, Maria Curie-Skłodowska University, Lublin, Poland

**Keywords:** Pressurized planar electrochromatography (PPEC), Electroosmotic flow velocity, Organic modifier, Zeta potential, Dielectric constant, Viscosity of the mobile phase

## Abstract

The aim of this work was to find a relationship between electroosmotic flow (EOF) velocity of the mobile phase in pressurized planar electrochromatography (PPEC) and physicochemical properties like zeta potential, dielectric constant, and viscosity of the mobile phase as well as its composition. The study included different types of organic modifiers (acetonitrile, methanol, ethanol, acetone, formamide, *N*-methylformamide and *N*,*N*-dimethylformamide) in the full concentration range. In all experiments, chromatographic glass plates HPTLC RP-18 W from Merck (Darmstadt) were used as a stationary phase. During the study we found that there is no linear correlation between EOF velocity of the mobile phase and single variables such as zeta potential or dielectric constant or viscosity. However, there is quite strong linear correlation between EOF velocity of the mobile phase and variable obtained by multiplying zeta potential of the stationary phase–mobile phase interface, by dielectric constant of the mobile phase solution and dividing by viscosity of the mobile phase. Therefore, it could be concluded that the PPEC system fulfilled the Helmholtz–Smoluchowski equation.

## Introduction

### Capillary Electromigration Techniques

Over the last decades growing interest of researchers in electromigration techniques can be seen. In the literature, there are increasing examples of their applications [especially capillary electrochromatography (CEC) and capillary electrophoresis (CE)] for efficient separation of the different components. In those techniques the mobile phase/buffer solution is driven into movement by the electric field. Many works have been published on electroosmotic flow (EOF) of the mobile phase in abovementioned techniques [1–5]. Much attention is devoted to the explanation of the mechanisms of the electroosmosis [5–8]. The EOF and especially its advantages are usually characterized [6–9]. Attention is also paid to the factors influencing its value [4–10]. The type of organic modifier plays an important role in the EOF generation [4, 6, 7, 9–11]. The linear velocity of the mobile phase flow is described by the following equation (Helmholtz–Smoluchowski equation):

1 $$ u_{\text{EOF}} = \varepsilon_{0} \varepsilon_{r} \zeta E/\eta $$

where *ε*_0_ is the electrical permittivity of vacuum, *ε*_*r*_ the dielectric constant of a liquid mobile phase, *ζ* the electrokinetic (zeta) potential, *E* the electric field strength and *η* the mobile phase viscosity.

According to the Helmholtz–Smoluchowski equation (1) the velocity of EOF is directly proportional to the dielectric constant and zeta potential and inversely related to the viscosity of the liquid. It is known that all variables from this equation are affected by organic modifier type and concentration [6]. Acetonitrile is the most commonly used solvent in CEC, because EOF values generated using systems containing this solvent are relatively high (about twice as high as for methanol and about three times as high as for tetrahydrofuran) [10]. In the majority of published papers, authors assumed that the zeta potential is constant, independent of the type and concentration of organic modifier in the mobile phase solution [4, 7, 9–11]. Therefore, the calculated value of ratio of the dielectric constant of the solvent to its viscosity should be directly proportional to the EOF [4, 6, 7, 9, 10]. However, experimental data show that this is not always true e.g. many authors observed unexpectedly high EOF velocities at high acetonitrile concentration [6, 7, 12]. The EOF dependence on the percentage of organic modifier in the mobile phase is also interesting. However, the resulting EOF values reported in the literature for acetonitrile as an organic modifier are contradictory. In some papers it was indicated that EOF increases with increasing concentrations of acetonitrile in the mobile phase, some results showed that EOF decreases with increasing concentrations of acetonitrile, but sometimes study showed that dependence of EOF versus percentage of acetonitrile has a minimum EOF at about 60–70 % of the organic modifier [10]. It should be noticed, that the operating conditions such as stationary phases, pH, temperature, electrolyte, ionic strength were not the same in the cited papers.

Cikalo et al. [9] had performed intensive investigation on open and laboratory packed fused-silica capillaries. The effect of acetonitrile content was studied over aqueous solutions, in the range 20–80 % (*v/v*) with phosphate electrolyte, pH 7.5 of 10 mM ionic strength when prepared in water. Four solvents were investigated: acetonitrile, methanol, acetone and 2-propanol. There is little difference between the EOF velocities in CE and CEC at over 40 % acetonitrile, and both techniques appear to follow the same trend. Experiments with acetone, acetonitrile and methanol as the mobile phase components show an EOF minimum in the range 50–70 % organic solvent. Similar results were found by Bartle and Myers [7]. Wright et al. [12] have also sub-witnessed this behavior for acetonitrile–water systems without supporting electrolyte. They have suggested it can be explained by changes in solvent polarity and hydrogen bond donor ability. However, Cikalo et al. [9] found that mixtures containing above 80 % acetonitrile were unable to support EOF.

Cahours et al. [11] performed investigation using phenyl silica and acetonitrile in the concentration range 30–80 % [Tris–HCl buffer (pH 8), ionic strength 5 mM)] in CEC. They showed that an increase of the organic modifier content induced an increase of EOF. The authors also noted that there were contradictory reports on this topic. Colon et al. [6] reported that in general it had been observed that with increased percentage of acetonitrile, the EOF velocity increased. Interesting finding has been described by Choudhary and Horvath [13]. They performed two sets of experiments: for the first one the ionic strength was not kept constant, for the second one, electrolyte concentration was kept constant. It was found that for CE experiments EOF velocity always decreased by increasing the organic modifier percentage. In CEC, however, keeping the electrolyte concentration constant, the EOF velocity increased with the percentage of acetonitrile.

Geiser et al. [4] have proposed new approach for the system with fused silica capillary (CE). They determined a correction factor for the *ζ* variation. The selected term was the donor number (DN) of a solvent. DN is a quantitative measure for a solvent’s ability to donate electrons, i.e. to bind a proton. Dividing term *ε*/*η* ratio by DN, a fully acceptable *r* ^2^ (EOF vs. *ε* DN *η* ^−1^) of 0.876 was obtained with all data (27 solvent types). Researchers have formulated following hypothesis: in the DN value differences in zeta potential of the systems with various solvents are involved.

### Planar Electrochromatography

Planar electrochromatography (PEC), which is performed in a three-phase system (gas, liquid and solid) and pressurized planar electrochromatography (PPEC), in which separation is performed in a two-phase system (liquid and solid) [14], are planar equivalents of the CEC. In the PPEC the mobile phase is also driven into movement by electric field contrary to conventional thin layer chromatography (TLC) where capillary forces are responsible for movement of the mobile phase. Forcing the flow of the mobile phase by electric field provides a number of important advantages of PPEC over TLC [14–30]. First of all the flat profile of the EOF makes the electrochromatographic separations more efficient compared to conventional TLC. Height of theoretical plates obtained in PPEC systems is very low [14, 19, 21, 23]. Also short time of separation process of solutes with PPEC technique is concerned with EOF velocity of the mobile phase (it is independent on particle diameter of the stationary phase of PPEC systems). This flow can be easily enhanced by increase of electric field strength [26]. Moreover, EOF velocity does not decrease with increase of development distance of electrochromatogram [24]. That is way PPEC enables to perform fast separation applying long distance of electrochromatogram development and high separation performance.

Different separation selectivity of PPEC systems relative to liquid chromatography ones is another advantage of PPEC. This feature is related to electrophoretic effect, which is involved in the separation process when charged solute molecules are in a separated sample. So, these all features make PPEC mode very attractive for the separation of different classes of mixture components especially of pharmaceutical and biomedical interest.

In planar electrochromatography systems, similarly as in other chromatographic systems, the flow rate of the mobile phase is important variable influencing separation efficiency. Therefore, it is important to understand the factors affecting EOF of the mobile phase. This aspect is particularly important to PEC systems where balance between liquid being driven to the surface of the adsorbent layer and mobile phase evaporation from the stationary phase surface should be obtained. Nurok et al. [31] have shown that pH, buffer concentration, and applied voltage significantly affect both EOF velocities (and also flow to the surface of the adsorbent layer) and Joule heating which enhances evaporation of liquid from the layer surface. In PPEC systems problems with flux and evaporation of the mobile phase have been eliminated, but the value of the flow rate of the mobile phase is still an important factor affecting separations efficiency. There are a few noteworthy papers, which deal with this subject including two of which were written by our group. In the first one the influence of polarization voltage, buffer concentration, pH and stationary phase type was investigated [18]. In the second paper we drew attention to other factors, such as prewetting procedure, temperature, and mobile phase composition. As it was stressed in the paper, there is no systematic investigation regarding the later factor. We showed two chromatograms, from which it follows that an increase of solvent strength, with increasing acetonitrile concentration in the mobile phase from 80 to 90 % can be observed [20]. Investigation regarding different factors affecting the EOF of the mobile phase was also performed by Nurok et al. [14, 16]. They reported results on examination of relationships migration distance of test solutes versus temperature, buffer concentration and pressure against adsorbent layer.

Therefore it should be noted that the influence of modifier type and its concentration on the EOF in PPEC systems have not been carried out so far. The main objective of this paper is to find relationship between mobile phase flow velocity and composition of the mobile phase, physical and chemical properties of the mobile phase in PPEC technique. Special attention is paid to the viscosity, dielectric constant and the zeta potential because the type and concentration of the organic modifier affect all of the above variables. Finding the right correlation between these variables will facilitate prediction of the approximate value of EOF velocity for a particular eluent. This could simplify the selection procedure of a suitable mobile phase, i.e. one that enables generation of the appropriate (i.e. high) flow velocity in PPEC system. It is evident that the composition of the mobile phase influences the separation selectivity. It is also known, that there are many other factors affecting the value of EOF, such as the applied voltage.

## Experimental

### Chemicals and Materials

Chromatographic glass plates HPTLC RP-18 W were supplied by Merck (Darmstadt, Germany). Chemicals and solvents were of analytical reagent grade. Acetonitrile, methanol, ethanol, acetone, *N*,*N*-dimethylformamide, acetic acid, sodium acetate were purchased from POCh (Gliwice, Poland). Citric acid monohydrate was supplied by Merck (Darmstadt, Germany). *N*-methylformamide, and formamide were supplied by Sigma-Aldrich (Steinheim, Germany), Sarsil W, Sarsil H-50 and hardener (utwardzacz W) were obtained from Silikony Polskie (Nowa Sarzyna, Poland). Bidistilled water was obtained in the laboratory.

### Buffer Solution and Mobile Phase Preparation

Mobile-phase solutions were prepared by mixing organic modifier with water and acetate acid buffer of pH 4.8 in appropriate proportion (*v/v*). The final buffer concentration in all mobile phase (with organic modifier) was 4.0 mM.

### Measurements of the Zeta Potential

Measurements of the zeta potential were performed with a Zetasizer Nano ZS manufactured by Malvern Instruments Ltd. (Malvern, Worcestershire, UK). Test sample was prepared by placing 0.01 g of the stationary phase scraped off HPTLC RP-18 chromatographic plate in a conical flask with ground-glass with solution of the mobile phase, composition identical as above (120 mL), and then the whole was subjected to sonication during 15 min. A sample was introduced into the apparatus with a syringe (20 mL). Considering the particle size (mean particle size 5–6 µm, particle size distribution 4–8 µm) and electrolyte concentration (4.0 mM) Smoluchowski’s approximation was selected for calculations.

## PPEC Experiments

### Chromatographic Plate Preparation

Chromatographic glass plates were cut into 10 cm × 2 cm pieces using TLC plate cutter (OM Laboratory Chigasaki, Japan), washed with methanol for 1 min and after drying in the air the plates were activated in an oven for 10 min. and left in desiccator for cooling. Then, 4 mm-wide margins were produced with sealant solution on the whole periphery of the adsorbent layer of each chromatographic plate. After that, the plate was dried in air and, next, in the oven at 105 °C for 60 min and left in desiccator for cooling. Detailed procedure description of the margin preparation is described in our previous papers [19, 20, 25]. The plates were used for experiments within 1 day. The next stage of the chromatographic plate preparation procedure was the prewetting the adsorbent layer of the chromatographic plate in the mobile phase solution for 2 min that was performed in special reservoir. According to our previous studies the prewetting time of the adsorbent layer is sufficient to obtain reproducible migration distances of separation solutes [15]. Detailed procedure of the chromatographic plate prewetting is described in the paper [19].

### Equipment for PPEC

**Fig. 1:**
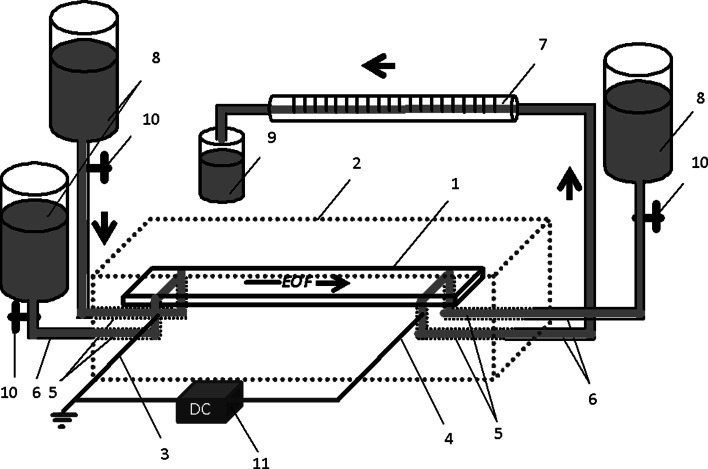
Conceptual view of the equipment for PPEC: *1* chromatographic plate, *2* body of the chamber, *3* anode, *4* cathode, *5* channel for the mobile phase, *6* Teflon tube, *7* 0.1 mL micropipet, *8* reservoirs, *9* waste, *10* valve and *11* high-voltage DC power supply with an ammeter; adapted from [18]

Device for PPEC was composed of the chamber for PPEC with the chromatographic plate, mobile-phase reservoirs, calibrated micropipet (100 µL) and high-voltage DC power supply, 10 kV, 120 W (Spellman, USA) with an ammeter and syringe pump, Kwapisz Duet 20/50 (Kwapisz, Warsaw, Poland). A conceptual view of the device for planar electrochromatography is presented in Fig. 1 (adapted from [18]).

### Operation of the Device for Pressurized Planar Electrochromatography

After the prewetting procedure, the chromatographic plate was immediately inserted into the PPEC chamber, and the chamber was closed with its lid. Afterwards the lid was pressed against the chromatographic plate, the channels and troughs in the chamber were filled with mobile-phase solution and then the voltage of an appropriate value was switched on (in all experiments potential equal to 2.0 kV was applied to electrodes). After a desired time necessary for performing the experiments, the voltage was switched off, and the chromatographic plate was taken out of the chamber and dried in air. Detailed description of the procedure of PPEC operation is presented in our previous paper [18]. The mobile phase from the cathode block was directed during the electrochromatography process to the calibrated micropipette. This enabled us to perform the measurement of the flow velocity of the mobile phase, which passed through the chromatographic plate during the process. This measurement was realized by control of the distance migration of the meniscus of the mobile phase (or air bubble injected) in the calibrated micropipette.

In all experiments, the chamber for PPEC was inserted in the Plexiglas cabinet to prevent the operator from coming in contact with equipment elements, which were under high voltage during the experiments.

All experiments were performed in triplicate.

It should be noted that the apparatus for PPEC was not temperature controlled. However, during the experiments the temperature was measured and the conditions of the experiment were chosen so that the changes in temperature were as small as possible (e.g. when 50 % of acetonitrile with 4 mM acetate acid buffer of pH 4.8 was used as the mobile phase and polarization voltage was equal to 2 kV—initial temperature was equal to 24.3 °C, after 2 min the temperature increased to about 25.0 °C and maintained this value until the end of the experiment (during next 8 min).

## Results and Discussion

Investigation of the relationship between the composition of the mobile phase and EOF velocity involved examination of impact of the type and concentration of the organic modifier. The study included the most commonly used solvents as acetonitrile, acetone, methanol, ethanol, formamide, *N*-methylformamide and *N*,*N*-dimethylformamide which are significantly different in physical and chemical properties.

The statistical data of EOF measurements are presented in Table 1. The repeatability of measurements was satisfactory for most solvents (RSD below 5 %). The largest scatters of the data were obtained for methanol and formamide (RSD over 8 % for three concentrations). The diversity of precision of measurements is likely due to small amount of repetitions of experiments (n = 5).

**Table 1:** Statistical data of EOF measurements

	Percentage of organic modifier (*v/v*)											
	0	10	20	30	40	50	60	70	80	90	96	98
ACN												
*x*	17.9	13.0		12.8		11.7	12.3	17.3	18.4	19.3		22.1
SD	0.17	0.65		0.34		0.35	0.14	1.18	0.86	0.43		0.69
RSD	0.98	5.03		2.65		3.00	1.13	6.83	4.68	2.22		3.11
CI	0.15	0.5		0.30		0.31	0.12	1.03	0.75	0.37		0.61
MeOH												
*x*			14.5		6.39		5.34		6.31			13.9
SD			0.69		0.68		0.51		0.56			0.31
RSD			4.74		10.6		9.59		8.94			2.24
CI			0.60		0.60		0.45		0.49			0.27
EtOH												
*x*			9.69		4.00		2.93		3.65		4.62	
SD			0.04		0.18		0.29		0.16		0.13	
RSD			0.44		4.61		9.79		4.44		2.80	
CI			0.04		0.16		0.25		0.14		0.11	
Acetone												
*x*			10.3		6.96		4.45		6.55			
SD			0.21		0.48		0.28		0.38			
RSD			2.03		6.96		6.24		5.84			
CI			0.18		0.42		0.24		0.34			
F												
*x*			38.5		67.3		65.1		56.9			35.7
SD			1.99		5.62		7.08		0.86			16.2
RSD			5.17		8.36		10.9		1.50			45.3
CI			1.75		4.93		6.20		0.75			14.2
NMF												
*x*			19.3		15.8		19.2		17.2			27.6
SD			2.81		1.28		0.76		0.38			1.93
RSD			14.2		8.07		3.96		2.22			6.99
CI			2.47		1.12		0.67		0.33			1.69
DMF												
*x*			9.37		6.67		5.02		5.64			12.2
SD			0.54		0.99		0.27		0.69			0.63
RSD			5.80		14.8		5.44		12.3			5.18
CI			0.48		0.87		0.24		0.61			0.55

*x* Average value (mm min^−1^), *SD* standard deviation, *RSD* relative standard deviation (%), *CI* confidence interval (*α* = 0.05), *ACN* acetonitrile, *MeOH* methanol, *EtOH* ethanol, *F* formamide, *NMF* *N*-methylformamide, *DMF* *N*,*N*-dimethylformamide

**Fig. 2:**
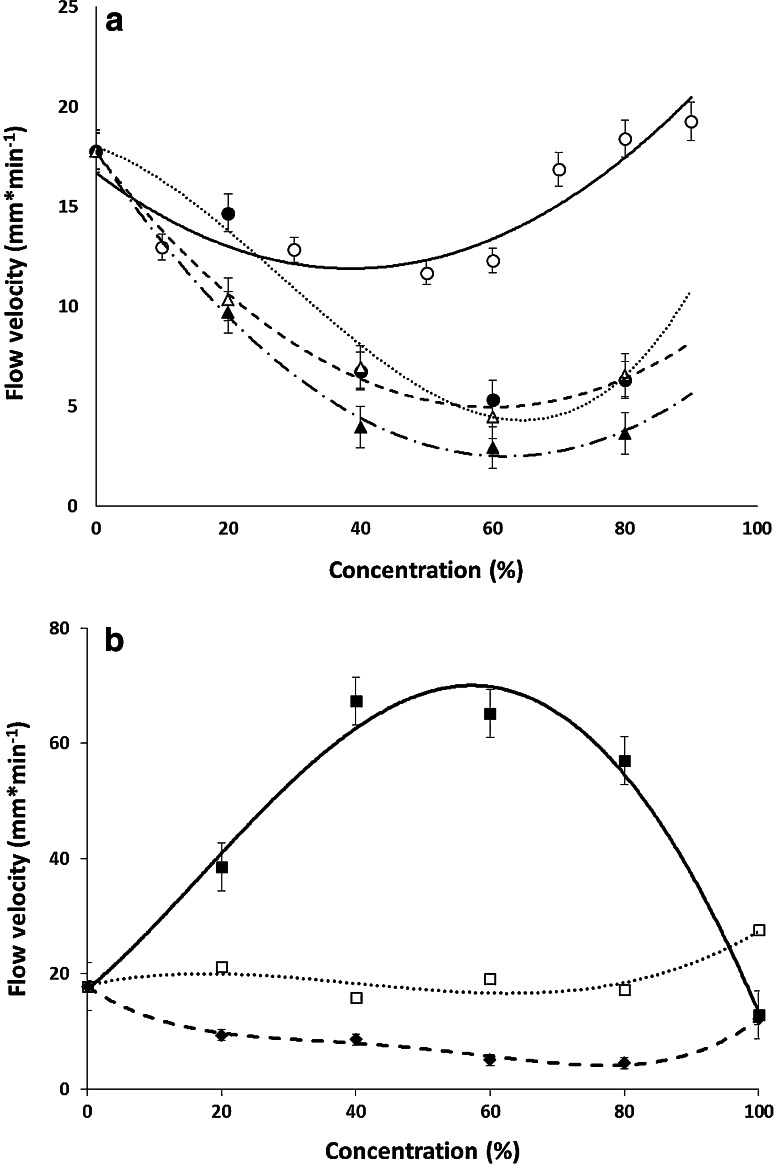
Electroosmotic mobile phase flow velocity dependent on concentration of the organic modifiers; HPTLC RP-18 W plate (Merck), potential = 2.0 kV; **a** acetonitrile *open circle*, methanol *filled circle*, acetone *open triangle* and ethanol *filled triangle*; **b** formamide *filled square*, *N*-methylformamide *open square* and *N*,*N*-dimethylformamide *filled diamond*

Obtained results are presented in the Fig. 2a, b as relationships EOF velocity of the mobile phase versus concentration, c, of the organic modifier. In these figures three types of relationships can be distinguished. Acetonitrile, acetone, methanol, and ethanol curves belong to the first type. These curves show evident minimum at the concentration range of 50–60 % *v/v* of the modifiers. The second type of relationship is characteristic for the mobile phase consisting of a mixture of water and dimethylformamide or methylformamide. Here small changes of the flow velocity of the mobile phase can be seen in the whole concentration range. Completely different course of changes in flow velocity of the mobile phase is shown for formamide system—the third relationship type. The curve *u*_EOF_ versus *c* shows a clear maximum at 40 % *v/v* formamide.

Our research has confirmed that the type of organic modifier shows significant influence on the EOF velocity generated in PPEC system and shown that its role is difficult to predict. It is well known that many variables are affected by change of the solvent type e.g. viscosity, dielectric constant and zeta potential. According to the Helmholtz–Smoluchowski equation, the velocity of EOF is directly proportional to the dielectric constant and electrokinetic potential of the solid–solution interface and inversely related to viscosity of a liquid solution. In this study, we attempted to determine the effect of each variable.

**Fig. 3:**
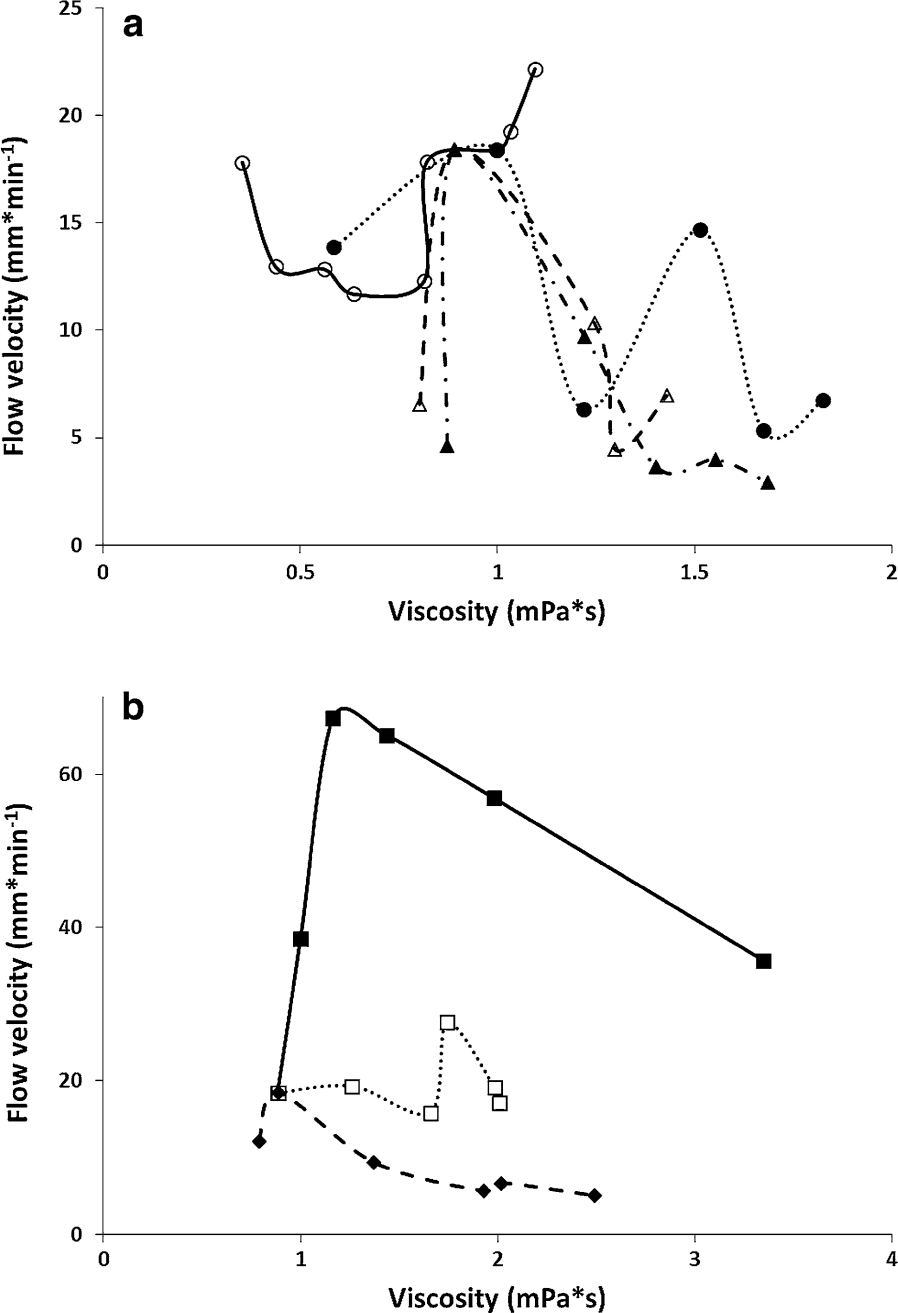
Electroosmotic flow velocity vs. viscosity of the mobile phase with the organic modifiers; **a** acetonitrile *open circle*, methanol *filled circle*, acetone *open triangle* and ethanol *filled triangle*; **b** formamide *filled square*, *N*-methylformamide *open square* and *N*,*N*-dimethylformamide *filled diamond*

If we consider the values of viscosity of the mixtures used and EOF velocity of the mobile phase, it can be seen that there is no simple correlation between these variables. Although the mobile phase solutions of the first relationship type (Fig. 2a) show indeed a flow velocity minimum in the curve *u*_EOF_ versus *c* and this minimum corresponds to the maximum value of the viscosity of these mixtures (40–60 %). A similar relationship cannot be observed for the mobile phase solutions, which belongs to the other relationship types. On the contrary, the mobile phase composed of formamide (Fig. 2b) has the highest value of the flow velocity at its highest viscosity. For the mobile phase from the second type the velocity of EOF of the mobile phase is almost constant for whole concentration range. To confirm these all observations graphs showing relationships between velocities of EOF versus viscosity of the mobile phase consisted of different type of organic modifier were prepared (Fig. 3a, b). For all solvents course of these relationships is very irregular. It could be concluded that the viscosity is not a sufficient parameter that enables estimating the value of EOF because there is no simple correlation between these variables. It should be noted that the viscosity values were taken from references [32–36], at 25 °C. This temperature is the closest to the temperature at which measurements of EOF were obtained (about 25 °C).

**Fig. 4:**
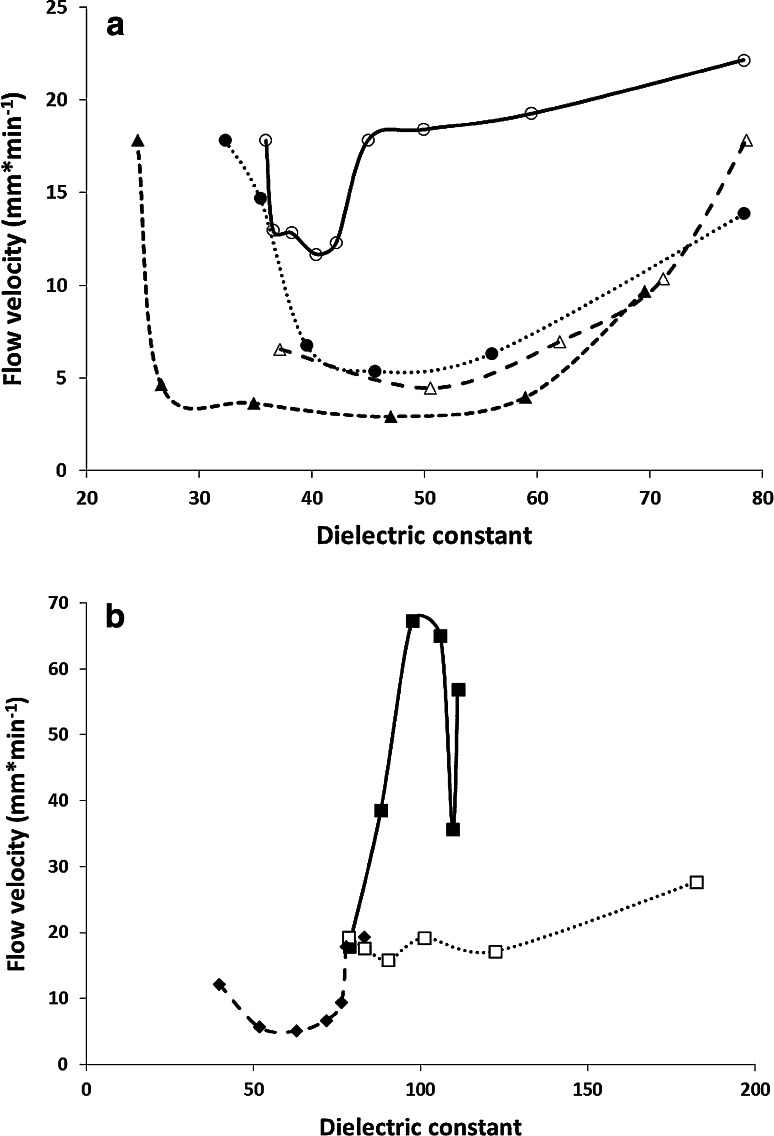
Velocity of the electroosmotic flow vs. dielectric constant of the mobile phase with the modifiers; **a** acetonitrile *open circle*, methanol *filled circle*, acetone *open triangle* and ethanol *filled triangle*; **b** formamide *filled square*, *N*-methylformamide *open square* and *N*,*N*-dimethylformamide *filled diamond*

Another variable that can affect the velocity of the EOF of the mobile phase is the dielectric constant, *ε*, of the mobile phase solution. In general, this parameter decreases with increasing concentration of the organic component of the mobile phase. Correlations of EOF velocity and dielectric constant of the mobile phase solutions are presented in Fig. 4a, b. The dielectric constant values were taken from references [32–36], at 25 °C [4, 37–41]. Buffer components were not included in the values of dielectric constants due to their constant and minor concentration. As it can be seen in these figures the correlation of the dielectric constant of the mobile phase solutions with the EOF velocity also did not allow for the clear identification of influence of this parameter on EOF in the systems investigated.

**Fig. 5:**
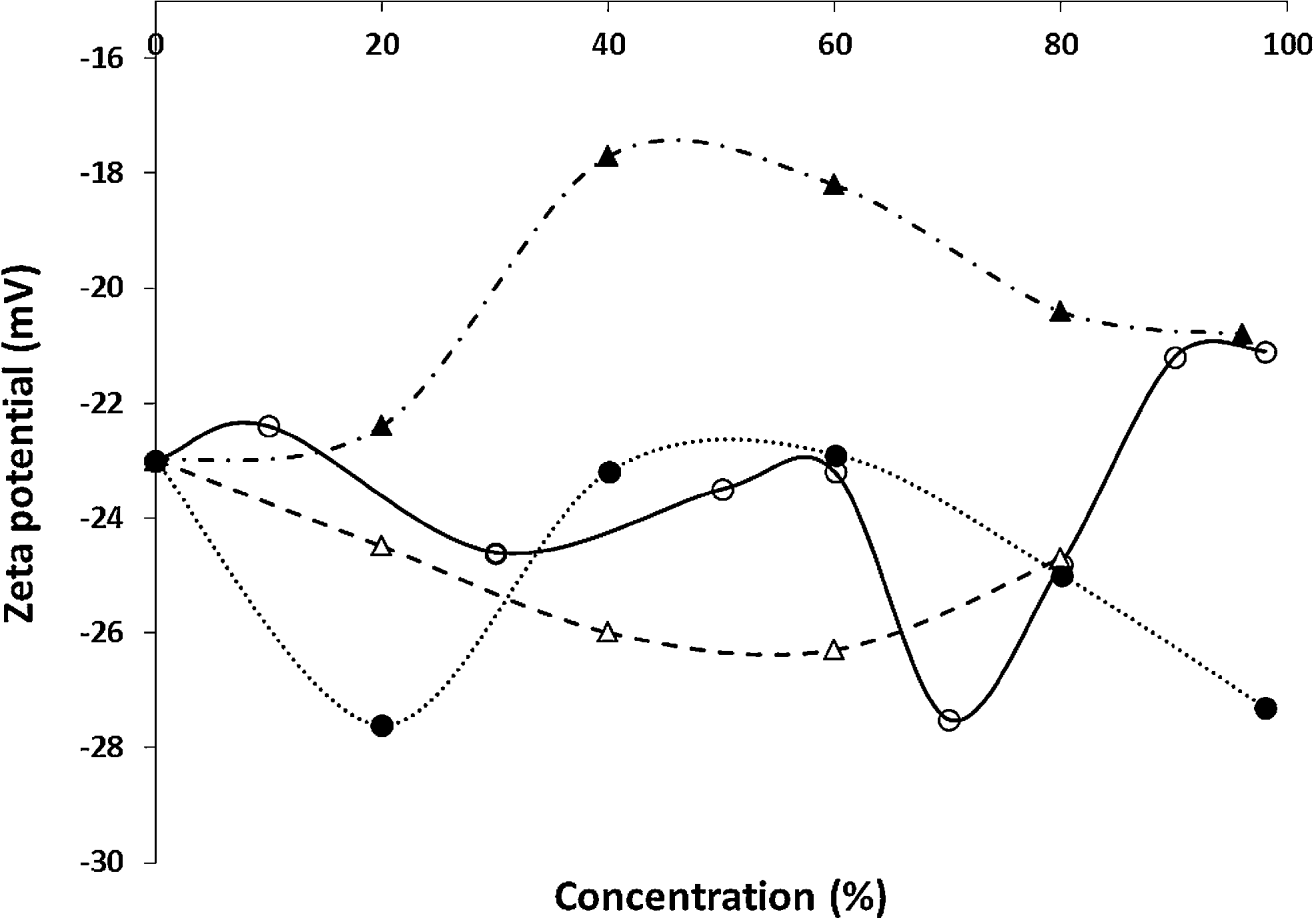
Zeta potential vs. concentration of the organic modifier (acetonitrile *open circle*, methanol *filled circle*, acetone *open triangle* and ethanol *filled triangle*) in the mobile phase in system with HPTLC RP-18 W chromatographic plate (Merck)

**Fig. 6:**
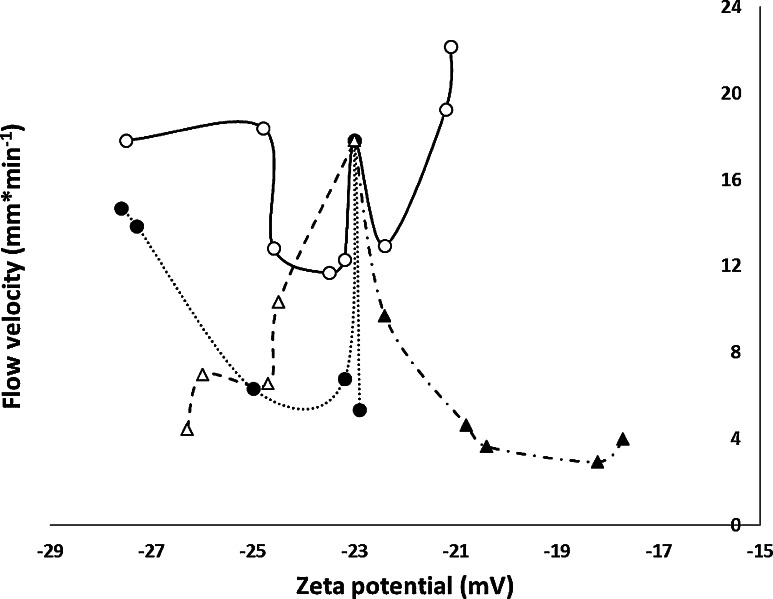
Electroosmotic flow velocity vs. zeta potential for acetonitrile *open circle*, methanol *filled circle*, acetone *open triangle* and ethanol *filled triangle* as organic modifiers

Subsequently the effect of the zeta potential was checked. In Fig. 5 the relationship of zeta potential versus percentage of organic modifier is presented. Software of our apparatus for the zeta potential measurements did not enable to measure zeta potential for solvent with a high dielectric constant (greater than a hundred). So data for formamides are not complete. However, due to the interesting properties of these solvents (high EOF) it was decided not to remove them entirely. The statistical data of zeta potential measurements are presented in Table 2. Precision of the measurements is satisfactory. For most solvents, RSD exceeds 5 % only for one concentration. As it can be seen, the relationship between the electrokinetic potential and the concentration of the organic modifier are irregular for all the systems investigated. Interestingly, the lowest negative value of zeta potential, specific for the systems containing over 90 % *v/v* of an organic component in the solution, corresponds to the highest value of EOF velocity of this solution as the mobile phase (Figs. 5, 6).

**Table 2:** Statistical data of zeta potential measurements

	Percentage of organic modifier (v/v)										
	10	20	30	40	50	60	70	80	90	96	98
ACN											
*x*	22.4		24.6		23.5	23.0	27.5	24.8	21.2		21.1
SD	0.93		0.91		1.34	1.30	1.27	0.92	0.38		0.29
RSD	4.15		3.68		5.71	5.64	4.61	3.71	1.78		1.36
CI	0.81		0.79		1.18	1.14	1.11	0.81	0.33		0.25
MeOH											
*x*		27.6		23.2		22.9		25.0			27.3
SD		0.68		1.47		0.85		0.71			1.09
RSD		2.48		6.31		3.69		2.83			3.99
CI		0.60		1.28		0.74		0.62			0.96
EtOH											
*x*		22.4		17.7		18.2		20.4		20.8	
SD		0.59		0.76		1.25		0.31		1.59	
RSD		2.62		4.30		6.85		1.53		7.62	
CI		0.51		0.67		1.09		0.27		1.39	
Acetone											
*x*		24.5		24.5		26.3		24.7			
SD		0.59		1.37		0.82		1.08			
RSD		2.41		5.60		3.10		4.37			
CI		0.52		1.20		0.72		0.95			

*x* Average value (mV), *SD* standard deviation, *RSD* relative standard deviation (%), *CI* confidence interval (*α* = 0.05), *ACN* acetonitrile, *MeOH* methanol, *EtOH* ethanol

In the next stage of the investigations three discussed variables (*ε*, *ζ*, and *η*) were considered as a single one, and it was denoted as *M*_D_. *M*_D_ resulted from multiplying the dielectric constant of the mobile phase by electrokinetic potential of the stationary phase–mobile phase interface and dividing by the viscosity of the mobile phase, i.e. *M*_D_ = *ε*_r_ *ζ* *η* ^−1^. Then Helmholtz–Smoluchowski equation (1) can be simplified to the following one:

2 $$ u_{\text{EOF}} = \varepsilon_{0} E M_{\text{D}} $$

**Fig. 7:**
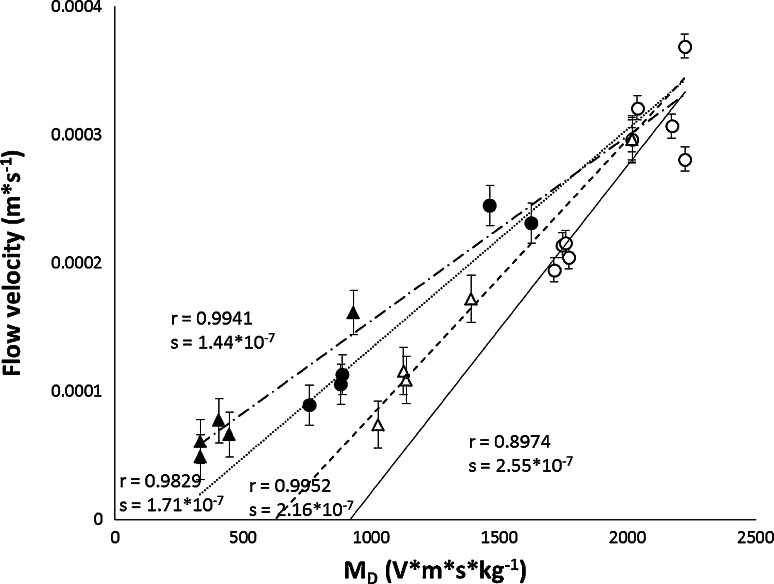
Electroosmotic flow velocity of the mobile phase vs. *M*_D_ (*M*_D_ = *ε* · *ζ* · *η* ^−1^); HPTLC RP-18 W chromatographic plate (Merck), potential = 2.0 kV; mobile phase comprised acetate buffer pH 4.8 (4 mM) and the organic modifiers (acetonitrile *open circle*, methanol *filled circle*, acetone *open triangle* and ethanol *filled triangle*)

Figure 7 shows the correlation between EOF velocity of the mobile phase and variable *Μ*_D_ for four binary mobile phase solutions with acetonitrile, acetone, ethanol, and methanol as organic modifiers (potential applied to electrodes was kept constant, 2.0 kV, see experimental section). As it can be seen, the EOF velocity of the mobile phase is directly proportional to the variable *M*_D_. The correlation coefficients of the above-mentioned dependences are very high (*r* above 0.98). Only for acetonitrile correlation is lower (*r* = 0.8974). The high value of the coefficients of correlation stands for additional confirmation of this relationship. Furthermore, the approximate value of the electric field strength is equal to 25 (kV m^−1^). If this value is multiplied by the value of the vacuum permittivity, *ε*_0_ = 8.854,187,817… × 10^−12^ (F m^−1^), it gives 2.21 × 10^−7^ (kg V^−1^ s^2^), which is approximately equal to the slopes of the plots obtained: *u*_EOF_ = *f*(*M*_D_) [slope ranges from 1.44 × 10^−7 ^(kg V^−1^ s^−1^) for ethanol to 2.55 × 10^−7 ^(kg V^−1^ s^−1^) for acetonitrile, compare Fig. 7.

Fairly high correlation of the variable *M*_D_ and the EOF velocity of the mobile phases investigated shows that the Helmholtz–Smoluchowski equation adequately describes PPEC systems in this regard. As it is apparent from the data presented in Fig. 7, based on the calculated value of *M*_D_ we can estimate the value of the EOF generated in the PPEC system. The highest flow velocities (from 11.7 to 22.1 mm min^−1^) have been obtained when acetonitrile was used as the organic modifier. Calculated *M*_D_ values are also the highest, and range 1,710–2,220 (V m s kg^−1^). It is noteworthy that the maximum EOF rate was obtained at a high concentration of the acetonitrile (98 %). For other solvents, the highest flow was obtained at high concentration of water (EOF for water was equal 17.9 mm min^−1^, and corresponding *M*_D_ is equal to 2,020). The lowest values of the flow velocity, from 2.93 to 9.69 (mm min^−1^), have been obtained when ethanol was used as the organic modifier and calculated *M*_D_ values show the lowest range amongst the obtained, from 330 to 1,390 (V m s kg^−1^).

## Conclusion

Our research has confirmed that similarly as in other electromigration techniques, the type and percentage of the organic modifier substantially affect the value of EOF in PPEC. We found that the relationship between the velocity of EOF of the mobile phase versus variable *M*_D_, which was obtained by multiplying dielectric constant of the mobile phase solution by zeta potential of the stationary phase–mobile phase interface and dividing by viscosity of the mobile phase solution, shows very good linear correlation that can be helpful to estimate the value of EOF of the mobile phase in PPEC systems. In addition, the data obtained confirm that the Helmholtz–Smoluchowski equation is valid for PPEC systems.
